# *Friesodielsia parvimitra* (Annonaceae), a New Species from Peninsular Thailand and a Note on *F. argentea*

**DOI:** 10.3390/plants13020189

**Published:** 2024-01-10

**Authors:** Jiratthi Satthaphorn, David M. Johnson, Charan Leeratiwong

**Affiliations:** 1 Center of Excellence for Ecoinformatics, School of Science, Walailak University, Thasala, Nakhon Si Thammarat 80161, Thailand; jiratthi.sa@wu.ac.th; 2 Department of Biological Science, Ohio Wesleyan University, Delaware, OH 43015, USA; dmjohnso@owu.edu; 3 Division of Biological Science, Faculty of Science, Prince of Songkla University, Hat Yai 90112, Songkhla, Thailand

**Keywords:** endemic, Flora of Thailand, ultramafic soil, Uvarieae, Yala

## Abstract

During botanical expeditions focused on the study of Annonaceae in Thailand, specimens resembling *Friesodielsia glauca* were collected from the lower part of peninsular Thailand but were believed to differ from that species in vegetative, flower, and fruit characters. A morphological investigation of the new specimens and species complex was conducted. Specimens (including types of *F. glauca* and similar species collected from Thailand and adjacent regions) were examined in the following herbaria: A, BK, BM, BKF, E, K, KEP, KKU, L, PSU, QBG, SING, and US. As a consequence, the new species *Friesodielsia parvimitra*, endemic to peninsular Thailand, is described and illustrated. The conservation status of the new species was provisionally assessed as Critically Endangered (CR, B2ab(iii)) following the IUCN’s guidelines. In addition, *F. argentea*, previously regarded *F. glauca*, is reinstated here as a distinct species based on several morphological differences and is reported as a new record from Thailand. Photographs, line drawings, and a revised key to *F. glauca* and similar species are presented.

## 1. Introduction

The genus *Friesodielsia* Steenis belongs to the family Annonaceae, subfamily Annonoideae, tribe Uvarieae, comprising approximately 50 species, distributed from India to Southeast Asia and New Guinea [1,2,3,4]. Plants of the genus are distinguishable by being woody climbers with fragrant flowers and short inner petals that are vaulted and connivent to form a dome over the center of the flower [5]. The genus is phylogenetically sister to *Dasymaschalon* (Hook.f. & Thomson) Dalla Torre & Harms and these two genera are sister to *Desmos* Lour., and the three genera together sister to *Monanthotaxis* Baill., based on nuclear and plastid markers [2,6,7]. These genera have shared morphological similarities, such as inaperturate pollen, parallel tertiary venation on the abaxial leaf surface, and *Friesodielsia* also shares basal leaf glands with *Desmos* and some species of *Monanthotaxis* [2,3,8].

Guo et al. [2], in a molecular phylogenetic study of the genus, reported that one subclade of *Friesodielsia* was characterized by flowers with three spreading outer petals and a flat petal base, whereas the other subclade has flowers with outer petals that are connivent before anthesis, concave at the base, and triquetrous toward the apex. One of the most widespread and variable species of the latter subclade is *F. glauca* (Hook.f. & Thomson) Steenis, which is distributed from peninsular Thailand to peninsular Malaysia and Borneo. Turner [9], in a review of climbing Annonaceae of Borneo, observed, however, that “There is considerable variation in the material here recognized under the name *Friesodielsia glauca*, both within Borneo and across its range. Some of the entities included appear quite distinct but intermediate forms occur”. A full list of currently recognized taxonomic synonyms was given by Turner [9].

During field surveys of Annonaceae in peninsular Thailand, we collected specimens of the *F. glauca* complex from several localities, including Waeng District (Narathiwat Province), Na Thawi District (Songkhla Province), and Betong District (Yala Province). However, all specimens differed consistently from typical *F. glauca* in several ways. Leeratiwong et al. [3] noted that some specimens collected from Songkhla Province may resemble *F. argentea* (J.Sinclair) Steenis, as they have broader outer petals and longer monocarp stipes. Another specimen, from Yala Province, was collected from a site at approximately 600 m above sea level, an unusually high elevation for *F. glauca*. In order to determine the taxonomic status of these collections, the following morphological study was conducted.

## 2. Taxonomic Results

The specimens previously identified as *F. glauca* from peninsular Thailand were distinguished into three sets, one corresponding to the typical *F. glauca*, another showing the features of *F. argentea*, currently included in *F. glauca*, and one that is interpretable as a species that is new to science, *F. parvimitra*.

**Figure 1:**
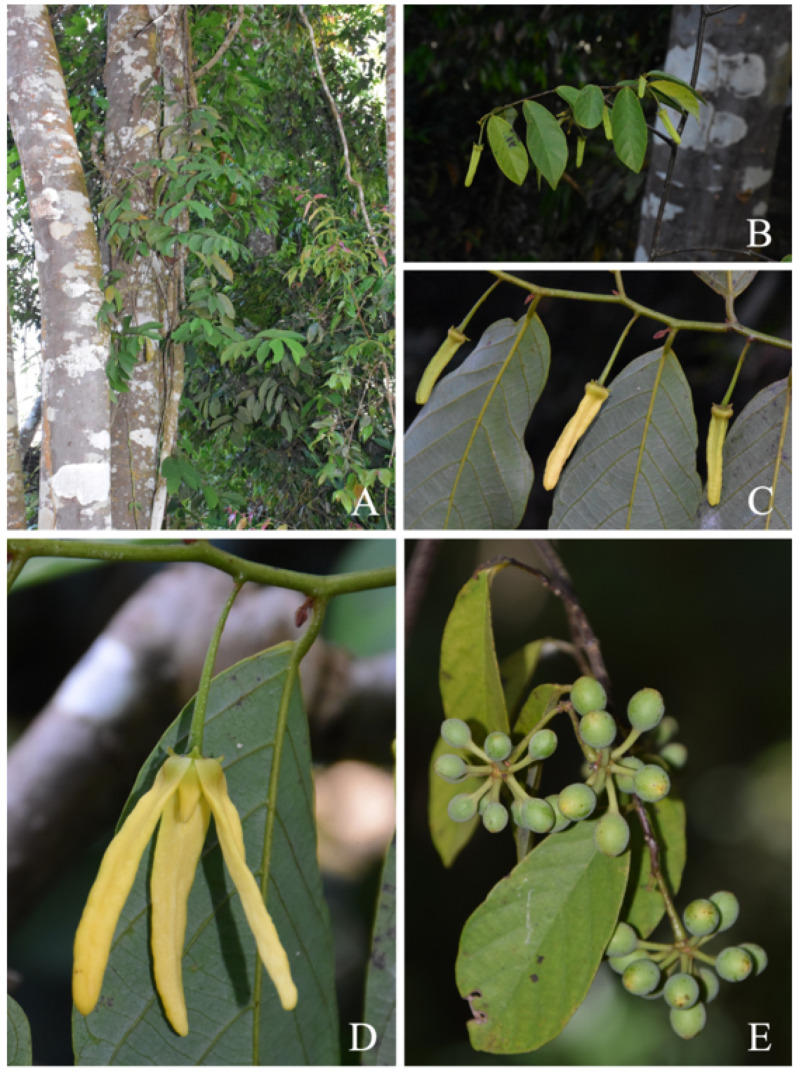
Photographs of *Friesodielsia parvimitra*. (**A**): habit, (**B**): flowering branch, (**C**): close-up of flowering branch with non-anthesis flowers, (**D**): flower at anthesis, and (**E**): fruits. Photos (**A**–**D**) by C. Leeratiwong, (**E**) by J. Satthaphorn.

**Figure 2:**
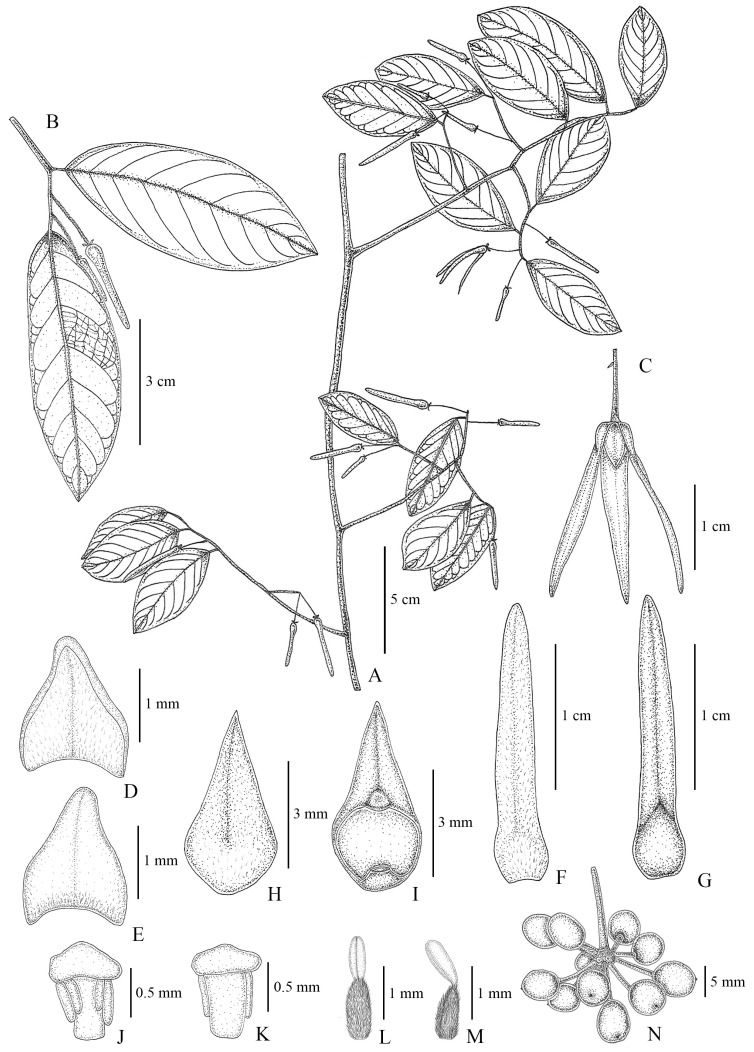
Line drawing of *Friesodielsia parvimitra*. (**A**): flowering branch, (**B**): close-up of flowering branch, (**C**): flower, (**D**): sepal (outside), (**E**): sepal (inside), (**F**): outer petal (outside), (**G**): outer petal (inside), (**H**): inner petal (outside), (**I**): inner petal (inside), (**J**): stamen (front), (**K**): stamen (back), (**L**,**M**): pistil, and (**N**): monocarps. All drawn by J. Satthaphorn.

*Friesodielsia parvimitra* Satthaphorn & Leerat., *sp. nov.* Figure 1 and Figure 2.

Type: Thailand, Yala Province: Betong, 9 March 2023, *Leeratiwong 23-2298* (holotype PSU; isotypes BKF, KKU, QBG).

Diagnosis: *Friesodielsia parvimitra* superficially resembles *F. glauca* in having inner petals shorter than half of the length of its outer petals, but differs in its non-glaucous lower leaf surface, much shorter flower pedicels (12–16 mm long vs. 20–40 mm long in *F. glauca*), shorter bracts (0.5–0.8 vs. 0.9–1.7 mm long), reduplicate sepals 2–3 mm long (vs. non-reduplicate sepals 4.0–5.5 mm long), and inner petals 4–5 mm long with puberulous appressed hairs on the outside surface (vs. 7–10 mm long with a glabrous outside surface).

Description: Woody climber, up to 15 m high. Twigs with brown-appressed hairs, becoming subglabrous when mature. Stem dark brown, with longitudinal ridges. Leaves subcoriaceous to chartaceous, elliptic to obovate-elliptic, 7–14 cm long, 3.5–6.0 cm wide, apex acuminate (acumen 2–15 mm long, about 2.9–10.7% of the total leaf length), base cuneate to rounded with conspicuous marginal glands, margin entire; upper surface dark green, with sparse whitish appressed hairs; lower surface pale green to whitish green, non-glaucous, with dense brown appressed hairs, the hairs denser on veins; secondary veins 6–9 on each side, yellowish; veinlets yellowish; petioles 3–10 mm long, flattened above with dense brown pubescence. Inflorescences internodal to leaf-opposed or terminal, 1(–3)-flowered; peduncles usually reduced, if present, up to 5 mm long; pedicels 12–16 mm long, slender, 0.3–0.4 mm wide, covered with dense yellowish appressed hairs, with a lanceolate or linear bract 0.5–0.8 mm long attached about 1/3 of pedicel length from the base. Sepals 3, yellowish green to green, valvate, reduplicate from below midpoint to the apex, coriaceous, ovate, 2–3 mm long, 1.0–1.2 mm wide, apex obtuse, venation obscure, outside covered with dense yellowish appressed hairs, inside glabrous, except along the margins. Petals in two series of 3, yellowish cream to yellow with pale green at base, valvate, free, coriaceous; outer petals spreading when mature, linear-oblong to linear-lanceolate, 20–25 mm long, 2–4 mm wide, triquetrous, apex obtuse, slightly concave on inner base, outside covered with dense yellowish appressed hairs, denser at base, inside glabrous; inner petals erect and coherent at margins in the distal third, 4–5 mm long, 2–3 mm wide (outer/inner petal length ratio = 5.0–8.3), apex acute, outside puberulous with appressed hairs only along midline, inside glabrous, sunken in the center. Stamens clavate, 1.0–1.2 mm long, anther dehiscence extrorse, apex of anther connective convex, thecae unequal. Carpels 20–30, oblong, 1.8–2.0 mm long, densely pubescent; stigmas falcate-capitate, cleft down the side. Immature monocarps 8–13, subglobose, obovoid, ellipsoid-obovoid or ovoid, 4–7 mm long, 6–7 mm wide, sparsely reddish-brown pubescent, apex rounded with apiculum ca 0.2 mm long, stipes 3–5 mm long, 0.8–1.0 mm wide. Seeds 1, ellipsoid-ovoid, 3–6 mm long, 4–5 mm wide, yellowish-brown, smooth.

Additional specimen examined: Thailand: Yala [Betong, 18 June 2023 (fr), *Leeratiwong & Satthaphorn 23-2326* (PSU)].

**Figure 3:**
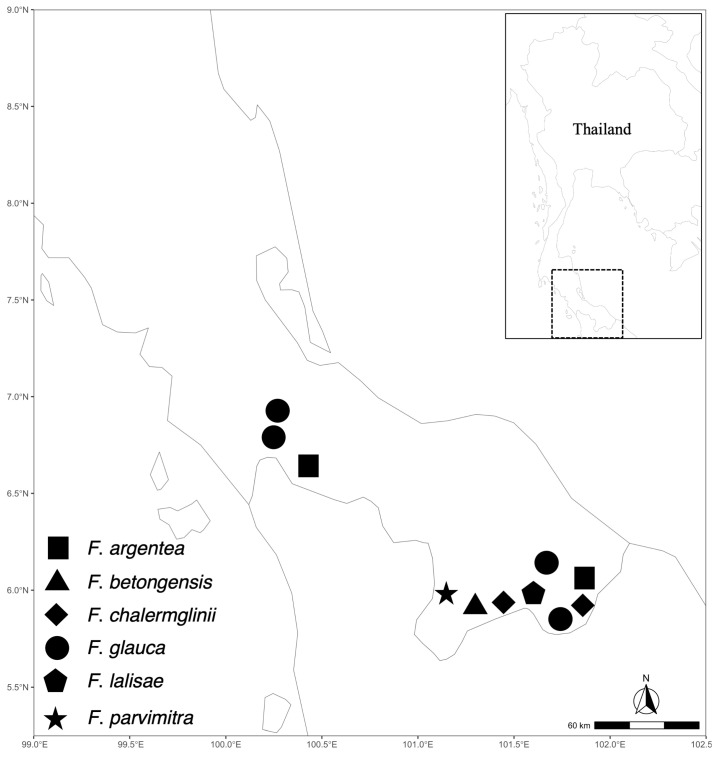
Distribution map of selected *Friesodielsia* species in lower peninsular Thailand.

Distribution: The plant is endemic to peninsular Thailand; it has only been observed in the evergreen forest in Betong District, Yala Province (Figure 3).

Ecology: The plant was found in a tropical rain forest in an open area on granitic and moist soil; alt. ca 600 m. From the field observation, this species exhibits a floral scent which is adaptively specialized to the pollination strategies of Annonaceae, as mentioned by Goodrich [10]. Within Annonaceae, flowers display a wide range of fragrance. Some species of *Friesodielsia* have a sweet-smelling scent, while others are stronger and more lemon-like.

IUCN conservation assessment: In Thailand, this species is represented by two collections from Betong District, Yala Province. From the field observations, the species has been found in only one location with approximately three individual plants and it is surrounded by para-rubber plantations and tourist attractions such as scenic viewpoints with deforestation for roads and accommodations. This observed disturbance may affect the quality of natural habitats in the future. As the species is found in a single locality, the area of occupancy is also less than 10 km^2^ (AOO = 4 km^2^), calculated by GeoCat [11]. The conservation status is here provisionally assessed following the IUCN’s guideline [12] as Critically Endangered (CR, B2ab(iii)) until more information about its population and possible threats in its natural habitat is obtained.

Vernacular: Bu nga klip lek (บุหงากลีบเล็ก).

Phenology: The plants flowers in March, and fruiting occurs in June.

Etymology: The specific epithet pertains to the inner petals that are connivent and short (less than 5 mm long), resembling a headdress.

Note: *Friesodielsia parvimitra* has inner petals that are much shorter than one-half the length of the outer petals and has slender pedicels which resemble those of *F. glauca*, but the differences between these two species are given in the diagnosis above and Table 1. We found that the new species occurs in the tropical rain forest at a higher elevation of approximately 600 m, while *F. glauca* occurs in lowland forests, up to 380 m [3]. The absence of a glaucous appearance on the lower leaf surface, and the fact that its outer petals separate at maturity, are characteristics that are similar to *F. lalisae* Damth., Baka & Chaowasku, but the new species is distinctly distinguishable by possessing much shorter inner petals (4–5 mm long vs. 19–25 mm long in *F. lalisae*), about four to five times shorter than the outer petals (Table 1). In addition, *F. lalisae* is found at a lower elevation, about 90 m [7].

**Figure 4:**
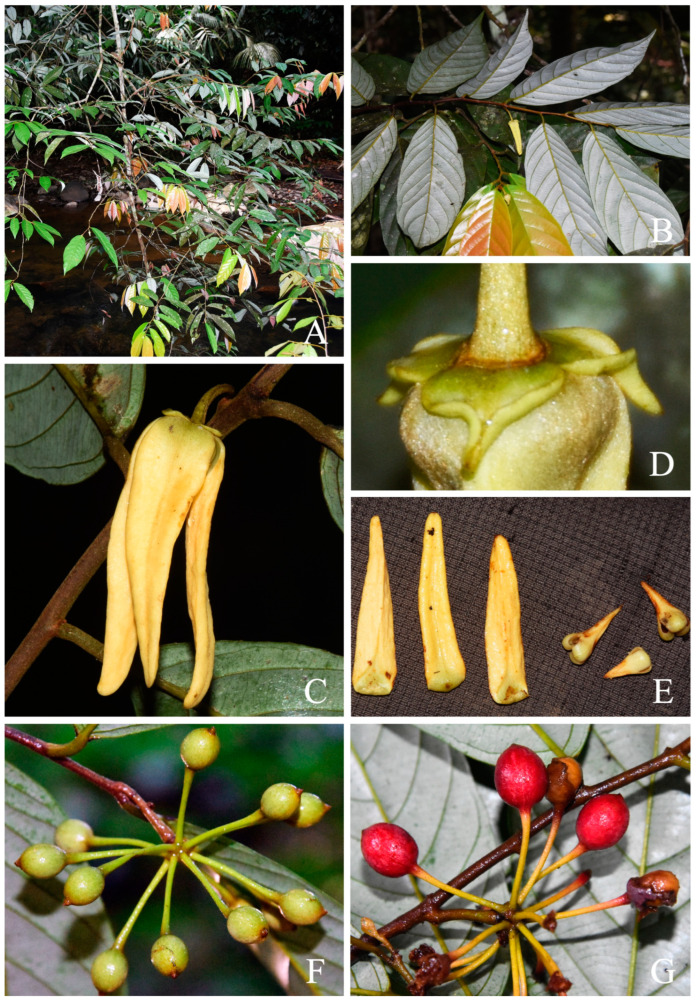
*Friesodielsia argentea* (J.Sinclair) Steenis: (**A**): habit; (**B**): flowering branch; (**C**): sepals of young flower, showing reduplicate margins; (**D**): mature flower; (**E**): outer (left) and inner (right) petals; (**F**): immature monocarps; (**G**): mature monocarps. Photos by C. Leeratiwong.

*Friesodielsia argentea* (J.Sinclair) Steenis, Blumea 12: 358. 1964.—*Oxymitra argentea* J.Sinclair, Gard. Bull. Singapore 14: 461. 1955. Figure 3 and Figure 4.

Type: Malaysia, Terengganu, Bukit Kajang, Kemaman, 14 November 1935, *Corner SFN 30457* (holotype: SING [SING0048676]; isotypes: K-2 sheets [K000691775 & K000691776], KEP).

Description: Woody climber up to 10 m high. Twigs covered with dense appressed pubescence. Leaves subcoriaceous to chartaceous, oblanceolate to elliptic, 10–20 cm long, 3.5–7.0 cm wide, apex acuminate to caudate (acumen 10–20 mm long, about 10% of the total leaf length), base broadly cuneate with conspicuous marginal glands; upper surface sparsely brown-red appressed pubescent; lower surface with moderate to sparse brown-red appressed hairs, silvery-glaucous; secondary veins 9–12 each side. Inflorescences internodal, 1-flowered, pedicels (12–)16–22 mm long, thick, 0.7–1.3 mm wide, covered with dense brown-pubescent, with a lanceolate bract, 2.0–2.5 mm long. Sepals 3, valvate, reduplicate from below midpoint to the apex, triangular-ovate, 4.5–6.0 mm long, 3.0–3.5 mm wide, apex long-acuminate, venation obscure. Petals in two series of 3, yellowish green to pale yellow; outer petals lanceolate-triangular to linear-lanceolate, 25–38 mm long, 4.5–6.0 mm wide, triquetrous, outside covered with appressed pubescence, inside glabrous; inner petals lanceolate, 9–15 mm long, 2.7–3.5 mm wide (outer/inner petal length ratio = 2.5–2.8), apex acuminate to caudate, both surfaces glabrous. Stamens clavate, 1.2–1.5 mm long. Carpels 28–32, oblong, 1.5–2.0 mm long, pubescent; stigmas globose, recurved. Monocarps (1–)5–20, ovoid or ellipsoid-ovoid, (8–)12–15 mm long, (6.5–)8–10 mm wide, smooth, verrucose when dried, covered with dense reddish-brown appressed hairs, stipes (15–)18–30 mm long, 0.7–1.5 mm wide, borne on pedicels, 13–20 mm long, 1.0–1.5 mm wide. Seeds 1, ellipsoid-ovoid to ovoid, 8.0–9.5 mm long, 6.5–7.0 mm wide, smooth.

Specimens examined: Thailand: Songkhla [Na Thawi, Khao Nam Khang National Park, 26 May 2021 (fl), *Leeratiwong 21-1732* (BKF, KKU, PSU), *ibid*., 17 June 2021 (fl), *Leeratiwong 21-1733* (BKF, KKU, PSU), *ibid*., 14 January 2022 (fr), *Leeratiwong 22-1812* (BKF, KKU, PSU), *ibid*., 25 February 2022 (fr), *Leeratiwong 22-1849* (PSU)]; Narathiwat [Waeng, 24 April 1972 (fr), *Sangkhachand et al. 1105* (BKF, L)].

Additional specimens examined: Malaysia: Selangor [Ulu Langat, Bukit Tangkol, Oct. (fr), *Millard KL 2204* (KEP), *ibid*., July (fr), *Millard KL 1587* (KEP), *ibid*., January (fr), *Millard KL 1351* (KEP)], Terengganu [Kemaman, Bukit Kajang, November (fl), *Corner SFN 30457* (KEP)], Perak [Goping (fl), *King’s Collector 4401* (K), *ibid*., *King’s Collector 4454* (K)].

Distribution: Peninsular Malaysia.

Ecology: Dry evergreen forest, along streams; alt. ca 150 m.

Vernacular: Bu nga bai ngueng (บุหงาใบเงิน) (Songkhla).

Phenology: The plants flowers from May to June, and fruiting occurs from January to April.

Note: *Friesodielsia argentea* differs from *F. glauca* in having shorter pedicels, longer bracts, broader pedicels and petals, more carpels and shorter monocarp stipes, as summarized in Table 1. Additional differences were found in leaf shape (oblanceolate to elliptic in *F. argentea* vs. obovate to oblanceolate-obovate in *F. glauca*) and the length of acumen (10–20 mm in *F. argentea*, about 10% of the total leaf length as opposed 6–20 mm in *F. glauca*, about 8.6–10% of the total leaf length). The collection *Sangkhachand et al. 1105* (BKF, L) was previously identified as *F. glauca* [5], but with better material, this collection could be correctly identified as belonging to *F. argentea*.

In contrast to the taxonomic treatment by Turner [9] according to which *F. argentea* was placed under *F. glauca* based on intermediate characteristics, this study supports the separation of these two species, since several specimens in peninsular Thailand and Malaysia revealed the morphological distinction of floral characteristics. *Friesodielsia argentea* is here resurrected and these findings support the discussion given by Leeratiwong et al. [3] and Damthongdee et al. [7].

**Figure 5:**
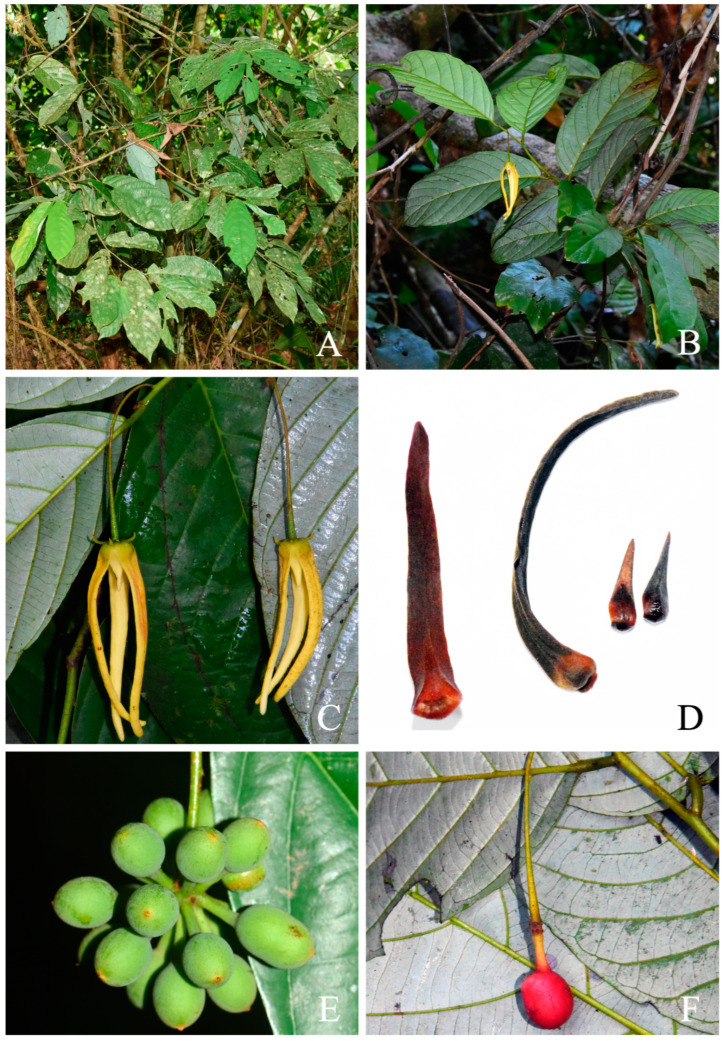
*Friesodielsia glauca* (Hook.f. & Thomson) Steenis: (**A**): habit; (**B**): flowering branch; (**C**): close-up of flowers; (**D**): outer (left) and inner (right) petals; (**E**): immature monocarps; (**F**): mature monocarp. Photographs by C. Leeratiwong (**A**,**B**,**D**–**F**) and A. Baka (**C**).

*Friesodielsia glauca* (Hook.f. & Thomson) Steenis, Blumea 12: 359. 1964; Leerat. et al., Thai For. Bull. (Bot.), 49(2): 225. 2021.—*Oxymitra glauca* Hook.f. & Thomson, Fl. Ind. 146. 1855; J.Sinclair, Gard. Bull. Singapore 14: 460. 1955.—*Richella glauca* (Hook.f. & Thomson) R. E. Fr. in Melchior, ed., Nat. Pflanzenfam., ed. 2, 17a(2): 139. 1959. Figure 3 and Figure 5.

Type: Malaysia, Prince of Wales Island [Penang], *Anonymous [W.E. Phillips] s.n.* (holotype K [K000691773]).

Description: Woody climber up to 7 m high. Twigs glabrous or with sparsely tomentose. Leaves membranous to chartaceous, mostly obovate to oblanceolate-obovate or rarely oblanceolate to ovate, 7–20 cm long, 3–12 cm wide, apex acuminate (acumen 6–20 mm long, about 8.6–10% of the total leaf length), base rounded to slightly cordate or rarely broadly cuneate, margin entire; upper surface pubescent when young, eventually glabrate; lower surface glabrate or with sparse reddish-brown appressed hairs, glaucous; secondary veins 8–16 each side. Inflorescences internodal to leaf-opposed, 1-flowered, pedicels (20–)25–40 mm long, slender, 0.3–0.8 mm wide, glabrous or covered with sparse reddish-brown appressed hairs, with a linear-lanceolate bract 0.9–1.7 mm long. Sepals 3, valvate, non-reduplicate, triangular, 4–6 mm long, 1.5–2.5 mm wide, apex acute, venation obscure, outside pubescent, inside glabrous. Petals in two series of 3, yellow; outer petals linear-lanceolate, (28–)30–45 mm long, 2.5–4.0 mm wide, triquetrous, apex obtuse to slightly acute, concave on inner base, midrib raised on outer surface, outside pubescent, inside glabrous; inner petals lanceolate-triangular, 7–10 mm long, 2.0–2.5 mm wide (outer/inner petal length ratio = 4.0–4.5), apex acuminate, both surfaces glabrous. Stamens clavate, 1.0–1.2 mm long. Carpels 15–18, cylindrical to oblong, pubescent; stigmas cylindrical. Monocarps up to 12, subglobose, obovoid, ellipsoid-obovoid or ovoid, 8–10 mm long, 6.5–9.0 mm wide, covered with sparse reddish-brown pubescence, stipes 5–8 mm long, 0.7–1.0 mm wide, borne on pedicels 25–50 mm long, 0.5–1.5 mm wide. Seeds 1, ellipsoid-ovoid, ca 7.5 mm long, 6 mm wide, smooth.

Specimens examined: Thailand: Songkhla [Hat Yai, Ton Nga Chang WS., alt. 250 m, 18 October 2021 (fr), *Leeratiwong 21-1851* (BKF); Sadao, Ton Nga Chang WS., Pha Dam forest station, 29 March 2022 (fl, fr), *Leeratiwong 22-1852* (BKF, KKU, PSU)]; Narathiwat [Cha Nae, Du Song Yo, 12 April 2020 (fl), *Leeratiwong 20-1538* (PSU), *ibid*., 26 April 2020 (fr), *Leeratiwong 20-1538* (PSU); Waeng, Hala-Bala W.S., 13 September 2003 (fr), alt. 380 m, *Promchua 53* (A, CMUB, L), *ibid*., 26 Aug. 2006 (fr), *Poopath 252* (BKF, A), *ibid*., 23 July 2008 (fr), *Puudjaa 1482* (BKF), *ibid*., 2 August 2020 (fr), *Leeratiwong 20-1850* (PSU)]. Malaysia: Sabah [Sepilok Forest Reserve, 7 May 1955 (fl), *Wood & Charington SAN16546* (L), Hutan Simpan Lumaku, Tenom, 10 September 1991 (fr), *Lantoh SAN132936* (L), Nabawan district, Syt Banawoods, logged area, Sungei Maadun, 26 June 1987 (fl), *Krispinus SAN119371* (L)], Sarawak [Kota forest near Sungei Telau, 20 October 1971 (fr), *Chai & Paie S27935* (L), Sungei Mayeng, Tau Range, 30 May 1956 (non-flowered), *Purseglove 5205* (L)], Negeri Sembilan [Pasoh Forest Reserve, 8 May 1996 (fl), *Gardette et al. E.G. 1807* (KEP, L)], Johor Bahru [Sungai Kayu, 11 October 1936 (fr), *Kiah SFN32044* (L)], Kedah [Sungei Ketil, 30 May 1931 (fl), *Henderson SFN24815* (KEP, SING)], Malacca [24 March 1867–68 (fl), *Maingay 3060* (K, KEP), *ibid*., 21 November 1867 (fl), *Maingay 3013* (K, KEP), *ibid*., *Maingay 58* (KEP)], Perak [Ulu or Nlu Kerling or Berling, April 1886 (fl), *King’s Collector 8853* (K, KEP), *ibid*., *King’s Collector 10192* (KEP)]. Singapore: Station 12½ mls Mandai Road, 16 October 1953 (non-flowered), *Sinclair 40044* (L, US). Indonesia: Kalimantan [Gunung Palung Nature Reserve, 15 June 1986 (fl), *Balgooy & Setten 5455* (L), Bukit Raya and upper Katingan (Mendawai) River area, 18 November 1982 (fl), *Mogea & Wilde 3517* (L)], Pakanbaru [Upper Riauw, 19 August 1960 (fr), *Soepadmo 117* (L)].

Distribution: Peninsular Malaysia (type), Singapore, Sumatra, Borneo.

Ecology: Primary seasonal evergreen hardwood forest or by streams in tropical rain forests, alt. 50–380 m.

Vernacular: Bu nga nuan paeng (บุหงานวลแป้ง).

Phenology: The plant flowers from March to April, and fruiting occurs from March to September.

## 3. Discussion

*Friesodielsia parvimitra*, the species new to science, exhibits some morphological similarities with its relative, *F. glauca*, which can also be found in peninsular Thailand, as outlined in the diagnosis above and Table 1. As *F. glauca* morphologically resembles *F. argentea*, the new species is also distinguishable from *F. argentea* by its lack of a glaucous lower leaf surface (vs. presence in *F. argentea*), smaller bracts that are 0.8–1.0 mm long (vs. 4–8 mm long), and shorter inner petals, 4–5 mm long (as opposed 9–15 mm long) (Table 1). The new species also appears to have similarities with *F. lalisae*; both lack a glaucous lower leaf surface, both have outer petals that separate at maturity, and both have a concave basal portion. However, *F. parvimitra* has longer pedicels, 12–16 mm long (vs. 9–10 mm long in *F. lalisae*), shorter outer petals, 20–25 mm long (vs. (34–)46–47 mm long in *F. lalisae*), and shorter inner petals, 4–5 mm long (as opposed 19–35 mm long in *F. lalisae*) (Table 1). The new species is also found in a tropical rain forest about 600 m above sea level, while its morphologically related species can be found in lowland forests, up to 380 m above sea level.

**Figure 6:**
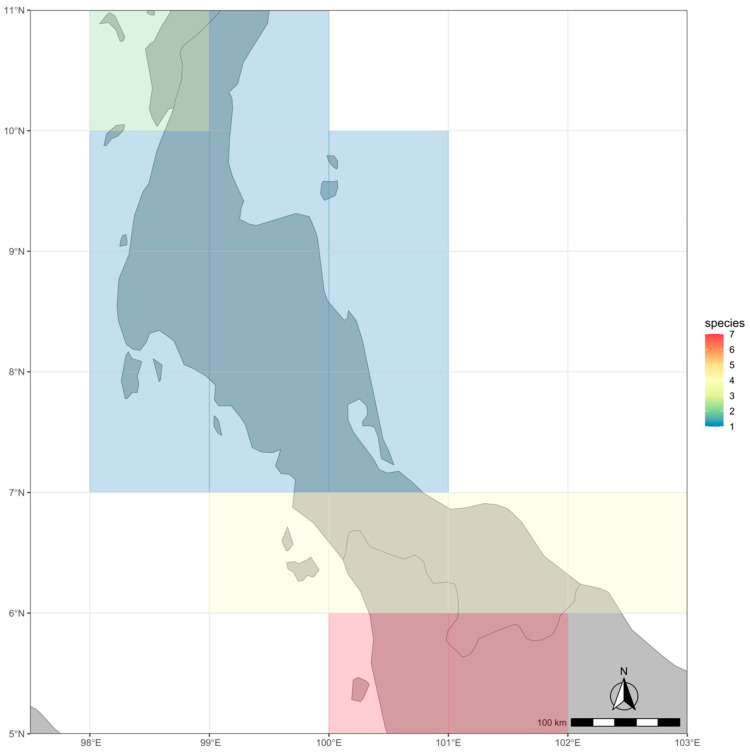
Density map of *Friesodielsia* in peninsular Thailand. The color labels on the map indicate species richness of *Friesodielsia species* from low (blue) to high (red).

*Friesodielsia parvimitra* is located in the forests near the Thai-Malaysian border. This range of forests encompasses several rare and newly described species, especially species within the family Annonaceae, which have been recently described, including *Goniothalamus roseipetalus* Leerat., Chalermglin & R.M.K. Saunders, *G. sukhirinensis* Leerat., Chalermglin & R.M.K. Saunders, *Polyalthia heliopetala* Leerat. & Bunchalee, *P. taweensis* Bunchalee & Leerat. and *Xylopia niyomdhamii* D.M. Johnson & N.A. Murray [3,13]. In addition, recent new species of *Friesodielsia*, including *F. betongensis* Leerat., *F. chalermgliniana* Leerat., and *F. lalisae*, were also discovered from this range of forests within Yala and Narathiwat Provinces [7,14]. Twenty of twenty-one *Friesodielsia* species can be found in peninsular Thailand, where the highest number of species of this genus is found, except for *F. discolor* (Craib) D. Das [3,7,14]. Of these, several species such as *F. betongensis*, *F. brevistipitata* Leerat., *F. chalermgliniana*, *F. khaoluangensis* Leerat. & Aongyong, *F. lalisae*, *F. longipetala* Leerat. & Chalermglin, *F. macrosepala* Leerat. & Aongyong, *F. phanganensis* Leerat., and *F. songkhlaensis* Leerat. are confined to peninsular Thailand as endemic species. The forest bordering Malaysia in the southernmost area of Narathiwat Province shows the highest number of species within a certain area (seven species, red blocks in Figure 6), while the number of species is slightly diminished at higher latitudes (Figure 6). These all recorded localities can be classified into tropical evergreen forest vegetation, in accordance with categories by Santisuk [15], while *F. khaoluangensis* establishes in the lower montane rain forest vegetation.

The new species discoveries in *Friesodielsia* and other Annonaceae genera in peninsular Thailand have largely resulted from recent expeditions to less explored areas. Several recorded locations are in the protected areas with a low level of human disturbance, which allows plants to thrive. Peninsular Thailand is also located in the overlapping zones of two major biodiversity hotspots in Asia, namely, Indo-Burma and Sundaland; these hotspots harbor a large number of endemic species [16] and are different from other regions of Thailand in that they have higher precipitation levels, since the area encounters monsoons throughout the year [17,18]. The additional abiotic factor that possibly drives the diversity in this area is the tropical ultramafic soil sites. This type of soil can be found in peninsular and northern Thailand, and in other regions in South and Southeast Asia, such as Pakistan, India, Myanmar, Vietnam, Malaysia, Indonesia, Philippines and also in other continents [19,20]. The ultramafic soil might serve as a selective pressure driving the speciation of species and providing suitably specific chemical and physical features in the soil–plant relations [20,21]. This finding supports several previous studies that show that various endemic species from different plant families are restricted to this type of soil, e.g., Campanulaceae, Euphorbiaceae, Orchidaceae, and Rosaceae [22,23,24,25,26].

Sinclair [27] distinguished *Oxymitra argentea* (=*F. argentea*) from *O. glauca* (=*F. glauca*) by noting that *Oxymitra argentea* has rusty-pubescent ellipsoid monocarps with distinctly longer monocarp stipes, as opposed to the glabrous globose monocarps of *O. glauca*. Further differences between *Oxymitra argentea* and *O. glauca* were given by Sinclair in the protologue of *O. argentea*: the pedicels of *O. glauca* were thicker, and the flowers were brownish yellow rather than yellow. The differences between these two species can be observed in Thai materials and selected specimens from the Malesian region; *F. argentea* has pedicels that are 0.7–1.3 mm thick, outer petals that are 4.5–6.0 mm wide, and monocarps with stipes that are (15–)18–30 mm long, while *F. glauca* has pedicels that are 0.3–0.8 mm thick, outer petals that are 2.5–4 mm wide, and monocarps with stipes that are 5–8 mm long (Table 1, Figure 4 and Figure 5). However, we found that the monocarp shape was more variable and overlapping between the two species, although the monocarps of *F. glauca* were generally larger than those of *F. argentea*. For these reasons, we conclude that *F. argentea* should be reinstated as a species distinct from *F. glauca*, and reported it for the first time from Thailand.

To update the additional knowledge for the Flora of Thailand, the revised key to species adapted from Leeratiwong et al. [3], including Thai *Friesodielsia* species that have outer petals that separate at maturity and triquetrous outer petals, is provided below.
**Revised key to the triquetrous-petaled *Friesodielsia* species in Thailand**1Pedicels equal or more than 59 mm long; twigs persistently hispid *F. filipes*1′Pedicels less than 59 mm long; twigs tomentose, pubescent, or glabrous 22Sepals 7–17 mm long 32′Sepals 2.5–6.0 mm long 53Inner petals about half length of outer petals; outer petals less than or equal to 17 mm long; pedicellar bracts apical *F. calycina*3′Inner petals much longer than half of outer petals; outer petals more than 17 mm long; pedicellar bracts at midpoint or basal 44Lower surface of leaves glaucous; bracts cordate to reniform; sepals with 5 distinct veins; carpels 7–15 *F. affinis*4′Lower surface of leaves non-glaucous; bracts broadly ovate; sepals with 5–7 obscure veins; carpels ca 30*F. chalermgliniana*5Inner petals longer or equal to half length of outer petals 65′Inner petals shorter than half length of outer petals 86Inner petals less than 18 mm long; pedicels of flower/fruit more than or equal to 14 mm long *F. betongensis*6′Inner petals equal or more than 18 mm long; pedicels of flower/fruit less than 14 mm long 77Outer petals 12–17 mm wide; inner petals 9–13 mm wide, apex acuminate; sepals crescent-shaped; leaves 7.5–15 cm wide, apex obtuse to rounded, occasionally emarginate or mucronate *F. latifolia*7′Outer petals 6–8 mm wide; inner petals 5.0–5.5 mm wide, apex acute; sepals transversely ovate; leaves 2.8–5.8 cm wide, apex more or less cuspidate, acute to acute-acuminate, rarely obtuse or rounded *F. lalisae*8Pedicels ca 10 mm long; leaves without brown-red appressed pubescence *F. khaoluangensis*8′Pedicels more than 10 mm long; leaves with brown-red appressed pubescence99Calyx 2–3 mm long; inner petals 4–5 mm long, puberulous outside; lower leaf surface non-glaucous*F. parvimitra*9′Calyx 4–6 mm long; inner petals 7–15 mm long, glabrous outside; lower leaf surface glaucous1010Pedicels 16–22 mm long, thickened; bracts 2.0–2.5 mm long; sepals 3.0–3.5 mm wide; monocarp stipe 15–18 mm long; sepals with margin reduplicate; leaves mostly oblanceolate to elliptic, acumen 10–20 mm long *F. argentea*10′Pedicels 20–40 mm long, slender; bracts 0.9–1.7 mm long; sepals 1.5–2.5 mm wide; monocarp stipe 5–12 mm long; sepals with margin non-reduplicate; leaves mostly obovate to oblanceolate-obovate, acumen 6–20 mm long *F. glauca*

## 4. Materials and Methods

The fieldwork was conducted in selected forest areas of the lower part of peninsular Thailand, namely, Waeng District (Narathiwat Province), Na Thawi District (Songkhla Province), and Betong District (Yala Province), from 2021 to 2023. We collected branches with flowers, which are the most important parts of the plants for circumscribing *Friesodielsia* species; we also returned to collect fruiting specimens. Herbarium specimens were made following the herbarium methodology proposed by Bridson & Forman [28]. We examined approximately 200 herbarium specimens in the following herbaria: A, BK, BM, BKF, E, K, KEP, KKU, L, PSU, QBG, SING, and US. The herbarium abbreviations follow the abbreviations proposed by Thiers [29]. We also examined digitized specimen images, available via Plants of the World online (http://powo.science.kew.org, accessed on 11 May 2023) and Global Plants on JSTOR (https://plants.jstor.org, accessed on 20 May 2023). All cited specimens in this study were observed by the authors and recently collected specimens were deposited at the PSU Herbarium. The literature regarding the genus *Friesodielsia* in Thailand and neighboring areas was reviewed [1,3,4,5,7,9,14,27,30,31].

Morphological evidence was used to circumscribe *Friesodielsia* species, following previous studies by Leeratiwong et al. [3,5,14] and Damthongdee et al. [7]. The collected specimens were compared with previously known species, based on both vegetative and reproductive parts, e.g., stems, leaves, inflorescences, flowers, and fruits, using a stereomicroscope (Olympus SZ51, Tokyo, Japan). Their morphological characteristics were described, and all plant terminology follows Beentje [32]. The morphological description was based on herbarium-dried specimens, while the colors of the specimens were derived from living materials [14]. The information for the IUCN conservation assessments following the IUCN’s guidelines [12] was collected using the online tool GeoCat [11]. The distribution map and the density map were constructed in Rstudio v3.6.3 [33] using the ggplot2 package [34] and its dependencies.

## 5. Conclusions

This study proposed *Friesodielsia parvimitra*, a species new to science, based on morphological evidence that clearly distinguishes it from the similar species *F. glauca* and *F. lalisae*. This new species can be provisionally assigned the conservation status of Critically Endangered (CR, B2ab(iii)) since the species is found in a single locality. *Friesodielsia argentea*, one of several synonyms of the complex species, *F. glauca*, is reinstated here as a distinct species based on the morphological characteristics of pedicel thickness, size of bract, petal width, and length of monocarp stipe. This is also a new record for Thailand. Our findings serve as additional knowledge for the Flora of Thailand. Up to now, twenty-one species of *Friesodielsia* have been recognized in Thailand. Of these, twenty species are found in peninsular Thailand, where the genus shows the highest number of species.

## Figures and Tables

**Table 1:** Comparison of diagnostic characters between *F. parvimitra* and closely related species.

Characters	*F. argentea*	*F. glauca*	*F. lalisae*	*F. parvimitra*
Glaucescence	Present	Present	Absent	Absent
Inflorescence	1-flowered	1-flowered	1-flowered	1–3-flowered
Pedicels	16–22 mm long, thick	20–40 mm long, slender	9–10 mm long, slender	12–16 mm long, slender
Bracts	2.0–2.5 mm long	0.9–1.7 mm long	unknown	0.5–0.8 mm long
Sepals	triangular-ovate, 4.5–6.0 × 3.0–3.5 mm, margin reduplicated	triangular, 4.0–5.5 × 1.5–2.5 mm, margin not reduplicated	broadly ovate, 3.0–3.1 mm long, margin not reduplicated	ovate, 2–3 mm long, margin reduplicated
Outer petals	25–38 × 4.5–6 mm	30–45 × 2.5–4 mm	(34–)46–47 × 6–8 mm	20–25 × 2–4 mm
Inner petals	9–15 × 2.7–3.5 mm, outside glabrous	7–10 × 2.0–2.5 mm, outside glabrous	19–25 mm long, outside puberulous with appressed hairs only along midline	4–5 × 2–3 mm, outside puberulous with appressed hairs
Carpels	28–32	15–18	ca 22	20–30
Stipe length	15–18 mm	5–18 mm	unknown	3–5 mm (immature)

## Data Availability

Data are contained within the article.
